# Tobacco Use Behaviors, Attitudes, and Demographic Characteristics of Tobacco Opinion Leaders and Their Followers: Twitter Analysis

**DOI:** 10.2196/12676

**Published:** 2019-06-04

**Authors:** Kar-Hai Chu, Anuja Majmundar, Jon-Patrick Allem, Daniel W Soto, Tess Boley Cruz, Jennifer B Unger

**Affiliations:** 1 University of Pittsburgh Pittsburgh, PA United States; 2 University of Southern California Los Angeles, CA United States

**Keywords:** tobacco, social media, online social networking, peer influence, social networking

## Abstract

**Background:**

Tobacco-related content on social media is generated and propagated by opinion leaders on the Web who disseminate messages to others in their network, including *followers*, who then continue to spread the information. Opinion leaders can exert powerful influences on their followers’ knowledge, attitudes, and behaviors; yet, little is known about the demographic characteristics and tobacco use behavior of tobacco opinion leaders on the Web and their followers, compared with general Twitter users.

**Objective:**

In this study, we hypothesized that opinion leaders use more tobacco products and have higher nicotine dependence than the other 2 groups (eg, followers and general Twitter users) and that followers—those who spread messages by opinion leaders—would more likely be in demographic groups that are vulnerable to tobacco marketing influence (eg, young adults and lower educational attainment).

**Methods:**

We constructed the social networks of people who tweet about tobacco and categorized them using a combination of social network and Twitter metrics. To understand the characteristics of tobacco opinion leaders and their followers, we conducted a survey of tobacco opinion leaders, their followers, and general Twitter users. The sample included 347 opinion leaders, 567 followers, and 519 general users. The opinion leaders had a median of 1000 followers, whereas followers and general users had fewer than 600 followers.

**Results:**

Opinion leaders were more likely than their followers to report past month use of tobacco products; followers, in turn, were more likely to report past month use of these products than general Twitter users. The followers appeared to be an especially vulnerable group; they tended to be younger (mean age 22.4 years) and have lower education compared with the opinion leaders and general users.

**Conclusions:**

Followers of Twitter tobacco opinion leaders are a vulnerable group that might benefit from antitobacco education to counter the protobacco communications they see on social media.

## Introduction

Social media has emerged as a popular forum for tobacco product users and prospective new users to learn about and discuss nicotine and tobacco products and for businesses to promote these products. Previously identified themes of tobacco-related social media posts include marketing and promotions by manufacturers, posts by tobacco users about their own tobacco experiences, discussions about flavors, and debates about tobacco product regulations [1-3]. Tobacco-related social media posts also contain content that could entice youth and nontobacco users to try tobacco products, including cartoons and other youth-oriented themes [4,5], hookah paired with alcohol in social settings [6], little cigar or cigarillos paired with marijuana [7], and pictures of people blowing large clouds of e-cigarette aerosol [8,9].

As social media messages about tobacco products can influence perceptions about the health effects and potential harms of these products and the social norms of use [10], it is important to understand as much as possible about these messages and the people who are disseminating them. Although previous research has focused on the content of tobacco-related messages on social media, less is known about the people who generate, receive, and propagate those messages. Previous analyses of information flow in Twitter have shown that a small number of *elite* users (approximately 20,000 people) generate nearly half of the tweets [11]. Only about 15.02% (1377/9165) of tweets received by ordinary users are directly from traditional mass media sources (eg, Cable News Network); most are filtered through opinion leaders who selectively retweet information from mass media sources [11]. Twitter opinion leaders—people who occupy central positions in their social networks, have numerous well-connected followers, have social status and credibility, and are emulated by their followers—are important members of Web-based communities as they control which information diffuses through social networks [12].

Opinion leaders discussing tobacco products on Twitter can potentially influence their followers to try new products, adopt beliefs about the relative harm of tobacco products, and support or oppose tobacco control policies. Thus, it is important to understand who these tobacco-related opinion leaders are and how their personal attitudes and behaviors might be influencing the discourse on the Web about tobacco products.

Intervention or education programs can benefit by leveraging opinion leaders to champion their ideas [13].

This study identified tobacco-related opinion leaders on Twitter by combining Twitter user metadata with techniques in social network analysis to develop a more comprehensive definition of opinion leaders. We then conducted surveys of these opinion leaders, people who follow these opinion leaders, and general Twitter users who are not engaged in tobacco-related discussions. We compared social network characteristics, demographic characteristics, tobacco product use, and nicotine dependence to identify differences among opinion leaders, followers, and general Twitter users. Opinion leaders, in this research, are operationalized as individuals strategically situated in their social networks. Their messages are disseminated widely via *shares* or *retweets* as they are viewed as subject matter experts. Research suggests that such high involvement and engagement in specific topics leads individuals to raise awareness about those topics and transition to polytobacco product use [14,15]. In keeping with this behavior, we hypothesized that opinion leaders would use more tobacco products and have higher nicotine dependence than the other 2 groups. We also hypothesized that followers, operationalized as individuals who predominantly follow and disseminate social media messages of opinion leaders, would more likely be in demographic groups that are vulnerable to the influence of tobacco marketing (eg, young adults and racial or ethnic minorities).

## Methods

### Data Collection

Twitter data were obtained with a custom Java 7 program based on Twitter4J v.4.0.3 that continuously accesses the Twitter streaming application programming interface (API) v.1.1 and collects tweets that contain any 1 of over 200 tobacco-related keywords, for example, cigarette, e-cigarette, or vape (see Multimedia Appendix 1). Twitter data were collected from March 2015 to March 2016. Along with the text of the tweet, the data include Twitter metadata such as the user name of the person who posted the tweet and whether the tweet was an original tweet or a retweet. This information was used to construct the retweet network by retrieving the data of every user who posted tobacco-related content.

Solely using Twitter metrics to identify opinion leaders can be misleading. Nontraditional accounts (eg, celebrities) can distort actual user classifications, whereas the number of followers is more likely to measure popularity rather than influence [16,17]. Therefore, we applied a combination of social network analysis–clustering algorithms and Twitter metrics to classify 3 types of individuals as follows: an opinion leader, a follower, and a general Twitter user. Opinion leaders and followers are additionally defined as users in our data who had posted tobacco-related content compared with general Twitter users who did not. First, a network was generated by linking users who had retweeted another user; this resulted in a retweet network defined by ties between the person who posted a tweet and the person who retweeted it. From this network, clusters were identified by conducting a modularity analysis. Modularity helps identify clusters within a network by grouping nodes (ie, Twitter users) that have more connections (ie, retweets) with others within a group than those outside of the group [18]. After the clusters were identified, opinion leaders were chosen as those who had been retweeted the most; followers were identified within each cluster as those who had retweeted others the most. Independently, general Twitter users were found by the Twitter API’s get-user-status function, which returns users who have recently posted a tweet about any topic (not just tobacco).

This method produced a convenience sample of 347 opinion leaders, 567 followers, and 519 general users. We sent Twitter private messages to potential participants inviting them to complete the survey. Each private message contained a unique, randomly generated link to a RedCap site where the survey was hosted. Clicking on the link identified the respondent as an opinion leader, follower, or general user who had been invited to complete the survey. This was done so that only people who received an invitation link could complete the survey. When respondents clicked on the link and arrived on the RedCap survey page, they saw an institutional review board–approved consent script. After clicking on a button indicating their consent to participate, they were directed to the survey. Participants received a US $20 gift card for completing the survey. Networks were constructed in April 2016. Surveys were sent out from May 2016 to June 2018.

### Measures

Participants self-reported their age, sex, race and ethnicity, and education. Social network characteristics were assessed by asking participants how many Twitter users they followed and how many Twitter users followed them. The survey asked which of the following products the participants had used in the past month: cigarettes, e-cigarettes, cigars, pipe tobacco, blunts, hookah, smokeless tobacco, cigarillo, marijuana, and alcohol.

### Statistical Analysis

The 3 groups (opinion leaders, followers, and general users) were compared on all measures, using analysis of variance for normally distributed continuous variables, the Kruskal-Wallis test for nonnormally distributed continuous variables, and chi-square for categorical variables.

## Results

### Demographic Differences Across Groups

The sample included 1433 completed surveys—347 opinion leaders, 567 followers, and 519 general users. The followers (mean age 22.4 years) were significantly younger than the opinion leaders (mean age 24.2 years) and the general users (mean age 25.2 years), *P*<.001. Compared with opinion leaders and general users, followers were more likely to be Hispanic (*P*=.03). General users were more likely than opinion leaders and followers to be African American (*P*=.03). Compared with opinion leaders and general users, followers had less education: only 11% (40/380) of followers had a bachelor’s degree or higher as compared with 19% (45/241) of opinion leaders and 26% (92/350) of general users (*P*<.001).

### Tobacco or Nicotine Product and Other Substance Use

For most of the tobacco products, opinion leaders reported the highest past month use prevalence, followed by followers and general users (Table 1). This pattern was evident for cigarettes, e-cigarettes, cigars, blunts, hookah, and cigarillos. Opinion leaders had the highest nicotine dependence scores, followed by followers and general users. Opinion leaders were also more likely than followers and general users to have used alcohol or marijuana in the past month.

**Table 1:** Comparison of opinion leaders, followers, and general users.

Twitter user characteristics		Opinion leaders (n=347)	Followers (n=567)	General users (n=519)	*P* value
**Social network size**					
	Number of Twitter users who follow the respondent (median)	1000	554	503	.001
	Number of Twitter users whom the respondent follows (median)	428	375	366	.01
Age (years)		24.2	22.4	25.2	<.001
Female, n/N (%)		122/242 (50.4)	211/392 (53.8)	202/360 (56.1)	.39
**Race and ethnicity, n/N (%)**					
	African American	26/242 (11)	40/392 (10)	67/360 (19)	.03
	Asian or Pacific Islander	15/242 (6)	21/392 (5)	14/360 (4)	.03
	Hispanic	51/242(21)	102/392 (26)	74/360 (21)	.03
	White	129/242 (53)	201/392 (51 )	176/360 (49)	.03
	Other	21/242 (9)	28/392 (7)	29/360 (8)	.03
**Education, n/N (%)**					
	High school or less	85/241 (35)	155/380 (41)	109/350 (31)	<.001
	Some college	111/241 (46)	185/380 (49)	149/350 (43)	<.001
	Bachelor’s degree or higher	45/241 (19)	40/380 (11)	92/350 (26)	<.001
Number of tobacco products used in past month (mean)		1.31	.98	.66	<.001
**Use of specific products in the past month, n/N (%)**					
	Cigarettes	93/347 (27)	117/567 (21)	83/519 (16)	.01
	E-cigarettes	65/347(19)	93/567 (16)	50/519 (10)	.01
	Cigar	30/34 (9)	37/567 (7)	23/519 (4)	.04
	Pipe	37/347 (11)	64/567 (11)	28/519 (6)	.01
	Blunt	96/347 (28)	118/567 (21)	73/519 (14)	.01
	Hookah	45/347 (13)	51/567 (9)	29/519 (6)	.01
	Smokeless	13/347 (4)	7/567 (1)	11/519 (2)	.04
	Cigarillo	60/347 (17)	67/567 (12)	41/519 (8)	.01
**Other substance use in the past month, n/N (%)**					
	Alcohol	159/229 (69)	221/378 (58)	215/354 (61)	.02
	Marijuana	103/219 (47)	142/371 (38)	96/330 (29)	<.001
Nicotine dependence score (mean)		1.48	1.18	1.02	.04
Number of tobacco brands followed on Twitter (mean)		.23	.18	.13	.11

## Discussion

### Principal Findings

Findings suggest that opinion leaders were more likely to report past month use of tobacco products than their followers; followers, in turn, were more likely to report past month use of these products than general Twitter users. The followers appeared to be an especially vulnerable group; they tended to be younger and have lower education. Opinion leaders had higher nicotine dependence scores and were more likely to report past month alcohol or marijuana use compared with followers and general users.

Tobacco opinion leaders on Twitter use a wide variety of tobacco products and other substances. As opinion leaders are typically held in high esteem by their followers, they play an important role in establishing and conveying social norms [19]. Opinion leaders who discuss their polytobacco and polysubstance use on Twitter might lead their followers to believe that these behaviors are normative, safe, or socially admirable. Followers, in turn, might emulate opinion leaders’ levels of tobacco use and become nicotine dependent.

Social media–based tobacco campaigns can address tobacco use disparities by tailoring messages that resonate with followers. Such focused efforts can potentially play an important role in educating followers who are typically younger and less educated than the other groups. Past evidence suggests that network-based interventions that involve identifying peer messengers result in improved health behaviors and more targeted delivery of interventions [13,20]. Social network analysis of a social media–based intervention also revealed that participants from vulnerable demographic groups (younger youth and females) may require additional outreach efforts [21]. Future tobacco health communication campaigns can take advantage of strategic delivery of health messages to followers on social media.

Solely using Twitter metrics to identify opinion leaders can be misleading, as bots, celebrities, and other nontraditional accounts can distort actual user classifications; Twitter metrics such as the number of followers are more likely to measure popularity rather than influence [16,17]. By using social network analysis in combination with Twitter metrics in this study, we are able to systematically identify emergent clusters in the Twitter tobacco network and then apply Twitter metrics to identify subgroup opinion leaders. This method helps prevent over-reliance on Twitter metrics such as follower or retweet count as the sole metric to define opinion leadership.

### Limitations

The study utilized a convenience sample by sending unsolicited messages to Twitter users. The tobacco opinion leaders, followers, and general Twitter users were selected on the basis of their positions in the Twitter social network; they had not previously expressed interest in participating in surveys. Twitter users who read their direct messages, click on a survey link, and complete a Web-based survey might not be representative of the general Twitter population; in addition, we have no method to verify that the user who takes the survey is the same as the original Twitter user who received the link.

### Conclusions

Despite these limitations, these findings provide important new information about people who disseminate and receive tobacco-related information on Twitter. Opinion leaders are influential as they occupy central positions in the social network and have the potential to communicate with a wide audience of Twitter users. Our findings indicate that tobacco opinion leaders use a wide variety of tobacco products as well as other substances. They may disseminate these attitudes to their Twitter followers who tend to be members of vulnerable populations (eg, young adults and lower educational attainment). Over time, repeated exposure to messages from tobacco opinion leaders could place followers at an increased risk for tobacco product experimentation and escalation. Although this survey was restricted to Twitter users aged 18 years and older, it is likely that younger Twitter users also follow tobacco opinion leaders, and these opinion leaders’ messages could persuade them to experiment with tobacco. This study demonstrates that it is possible to identify tobacco opinion leaders on Twitter and their followers and opens the opportunity to apply other methods of supplementing Twitter measures to classify Twitter users. Opinion leaders on the Web have large, well-connected social networks of social media users who may look to them for information, opinions, and advice. If the information disseminated by opinion leaders on the Web is incorrect or biased, their followers could make important decisions based on faulty information. Future research should determine how opinion leaders influence their followers’ offline tobacco behaviors.

## Appendix

Multimedia Appendix 1 List of tobacco-related keywords.

## Abbreviations

API: application programming interface
FDA: Food and Drug Administration
NIH: National Institutes of Health
