# Supplementary material for: Tobacco Use Behaviors, Attitudes, and Demographic Characteristics of Tobacco Opinion Leaders and Their Followers: Twitter Analysis
Source: J Med Internet Res. 2019 Jun 4;21(6):e12676. doi: 10.2196/12676 (PMC6746100; doi:10.2196/12676)
Supplement: Multimedia Appendix 1 [file jmir_v21i6e12676_app1.docx]

**Multimedia Appendix 1 List of Tobacco-Related Keywords**

| ecig | joye 510 | still blowing smoke | cigarillo |
| --- | --- | --- | --- |
| e-cigs | joye510 | notblowingsmoke | blunt |
| ecigs | joyetech | not blowing smoke | vaporcade |
| e-cigarette | lavatube | capublichealth | narguile |
| ecigarette | lavatubes | tobaccofreekids | shisha |
| e-cigarettes | logicecig | notareplacement | marlboro |
| ecigarettes | logicecigs | trulyfree | vuse |
| vape | smartsmoker | truly free | swisher |
| vaper | smokestiks | sb140 | black and mild |
| vaping | v2 cig | sb 140 | copenhagen |
| vapes | v2 cigs | sb24 | camel |
| vapers | v2cigs | sb 24 | snus |
| nicotine | v2cig | cherry tip cigarillos | pall mall |
| tobacco | mistic | mini-cigarillos | newport |
| cigarette | 21st century smoke | tip cigarillos | wakeup |
| cigarettes | logic black label | king edward cigars | cheerupbigtobacco |
| cigar | finiti | royal gold cigars | transformtobacco |
| atomizer | nicotek | sweet coronella | swishersweets |
| atomizers | cigirex | swisher blk | swisherartistproject |
| cartomizer | logic platinum | swisher sweets | swisherartistgrant |
| cartomizers | cigalectric | vapercon | swisheratl |
| ehookah | xhale o2 | vapercon west | blunation |
| e-hookah | cig2o | grimmgreen | blucigs |
| ejuice | green smart living | vapor | justyouandblu |
| ejuices | krave | electronic cigarette | plusworks |
| e-juice | secondhand vape | vape meet | thisfreelife |
| e-juices | secondhand vaping | EcigsSaveLive | swishersweeties |
| eliquid | second-hand vape | EcigsSaveLives | swishermusiccity |
| eliquids | second-hand vaping | EcigsSavesLives | tobacco21 |
| e-liquid | vape smoke | vapecon | sbx27 |
| e-liquids | ecig smoke | fresh empire | sbx25 |
| blu | e-cig smoke | freshempire | FDAdeeming |
| njoy | e-cigarette smoke | camel crush bold | FDAtobacco |
| green smoke | vape shs | camelcrushbold | e-smoke |
| south beach smoke | ecig shs | menthol | stillblowingsmoke |
| eversmoke | vape secondhand smoke | clove | vape second-hand smoke |
| hookah | esmoke |  |  |
